# Covered Stent Correction for Sinus Venosus Atrial Septal Defects, an Emerging Alternative to Surgical Repair: Results of an International Registry

**DOI:** 10.1161/CIRCULATIONAHA.124.070271

**Published:** 2024-12-19

**Authors:** Eric Rosenthal, Shakeel A. Qureshi, Kothandam Sivakumar, Matthew Jones, San-Fui Yong, Saleha Kabir, Pramod Sagar, Puthiyedath Thejaswi, Sebastien Hascoet, Clement Batteux, Younes Boudjemline, Ziyad M. Hijazi, Jamil A. Aboulhosn, Daniel S. Levi, Morris M. Salem, Edwin Francis, Aleksander Kempny, Alain Fraisse, Carles Bautista-Rodriguez, Kevin Walsh, Damien Kenny, Brian Traynor, Salim N. Al Maskari, James R. Bentham, László Környei, Muthukumaran C. Sivaprakasam, Ata Firouzi, Zahra Khajali, Lee Benson, Mark Osten, Alban-Elouen Baruteau, Matthew A. Crystal, Thomas J. Forbes, Stanimir Georgiev, Horst Sievert, Do Nguyen Tin, Daniel Springmuller, Anand Subramanian, Hussein A.M. Abdullah, Radwa Bedair, Francisco Chamié, Ahmet Celebi, Jesus Damsky Barbosa, Pieter De Meester, Luca Giugno, Zakaria Jalal, Clement Karsenty, Anastasia Schleiger, Gregory Fleming, Andre Jakob, Tevfik Karagoaz, Gur Mainzer, Gareth J. Morgan, Nazmi Narin, Shabana Shahanavaz, Zachary L. Steinberg, Osamah Aldoss, Elnur Alizade, Oliver Aregullin, Hélène Bouvaist, Thilo Fleck, Francois Godart, Sophie Malekzadeh-Milani, Paulo Motta, Angel Sanchez-Recalde, Juan Pablo Sandoval, Weiyi Tan, John Thomson, Pablo Tomé Teixeirense, Evan M. Zahn

**Affiliations:** 1 Paediatric and Adult Congenital Heart Disease, Evelina London Children’s Hospital, Guy’s & St Thomas’ Hospital Trust, UK (E.R., S.A.Q., M.J., S.-F.Y., S.K.).; 2 Department of Pediatric Cardiology, Institute of Cardio Vascular Diseases, Madras Medical Mission, Chennai, India (K.S., P.S., P. Thejaswi).; 3 Department of Congenital Heart Diseases, Centre de Reference Cardiopathies Congenitales Complexes M3C, Hospital Marie Lannelongue, Groupe Hospitalier Paris, Saint Joseph, Universite Paris-Saclay, Paris, France (S.H., C.B.).; 4 Sidra Heart Center, Sidra Medicine, Doha, Qatar (Y.B., Z.M.H.).; 5 Division of Cardiology, Ahmanson/UCLA Adult Congenital Heart Disease Center, Los Angeles, CA (J.A.A.).; 6 Division of Pediatric Cardiology, Mattel Children’s Hospital at UCLA, Los Angeles, CA (D.S.L., M.M.S.).; 7 Aster Medcity Hospital, Kochi, India (E.F.).; 8 Adult Congenital Heart Disease, Royal Brompton Hospital, London, UK (A.K., A.F., C.B.-R.).; 9 Department of Cardiology, Mater Misericordiae University Hospital, Dublin, Ireland (K.W., D.K., B.T.).; 10 Paediatric Cardiology, National Heart Centre, Muscat, Oman (S.N.A.M.).; 11 Department of Congenital Cardiology, Leeds General Infirmary, UK (J.R.B.).; 12 Gottsegen National Cardiovascular Center, Budapest, Hungary (L.K.).; 13 Department of Paediatric Cardiology, Apollo Children’s Hospital, Chennai, India (M.C.S.).; 14 Cardiovascular Intervention Research Center, Rajaie Cardiovascular Medical and Research Institute, Iran University of Medical Sciences, Tehran (A.F., Z.K.).; 15 Labatt Family Heart Center, Hospital for Sick Children, Toronto General Hospital, University of Toronto School of Medicine, ON, Canada (L.B., M.O.).; 16 Nantes Université, CHU Nantes, Department of Pediatric Cardiology and Pediatric Cardiac Surgery, FHU PRECICARE, France (A.-E.B.).; 17 Congenital Interventional Catheterization, Columbia University Vagelos College of Physicians and Surgeons, Morgan Stanley Children’s Hospital of New York–Presbyterian, New York (M.A.C.).; 18 Joe DiMaggio Children’s Hospital, Hollywood, FL (T.J.F.).; 19 Department of Congenital Heart Diseases and Pediatric Cardiology, German Heart Center, Munich, Germany (S.G.).; 20 CardioVascular Center Frankfurt, Germany (H.S.).; 21 Department of Pediatrics, University of Medicine and Pharmacy, Ho Chi Minh City, Vietnam (T.D.N.).; 22 Departamento de Cardiología Pediátrica, División de Pediatría, Facultad de Medicina, Pontificia Universidad Católica de Chile, Unidad de Cardiopatías Congénitas del Adulto, Instituto Nacional del Tórax, Santiago, Chile (D.S.).; 23 Pediatric Cardiology, Sri Jayadeva Institute of Cardiovascular Sciences, Bangalore, India (A.S.).; 24 Ibn- Albitar Center for Cardiac Surgery, Baghdad, Iraq (H.A.M.A.).; 25 Adult Congenital Cardiology, Bristol Heart Institute, UK (R.B.).; 26 INTERCAT–Interventional Cardiology, Rio de Janeiro, Brazil (F.C.).; 27 Department of Pediatric Cardiology, Dr Siyami Ersek Hospital for Cardiology and Cardiovascular Surgery, Istanbul, Turkey (A.C.).; 28 Cardiology and Hemodynamics, Pedro de Elizalde Children’s Hospital, Buenos Aires, Argentina (J.D.B.).; 29 Division of Congenital and Structural Cardiology, UZ Leuven and Department of Cardiovascular Sciences, KU Leuven, Belgium (P.D.M.).; 30 IRCCS Policlinico San Donato, Milano, Italy (L.G.).; 31 Department of Pediatric and Adult Congenital Cardiology, University Hospital of Bordeaux, France (Z.J.).; 32 LIRYC Electrophysiology and Heart Modeling Institute, Fondation Bordeaux Université, France (Z.J.).; 33 INSERM, Centre de recherche Cardio-Thoracique de Bordeaux, Pessac, France (Z.J.).; 34 Pediatric and Congenital Cardiology, Children’s Hospital CHU Toulouse, Institut des Maladies Métaboliques et Cardiovasculaires, Université de Toulouse, Institut National de la Santé et de la Recherche Médicale (INSERM), U1048, Clinique Pasteur, France (C.K.).; 35 Department of Congenital Heart Disease–Pediatric Cardiology, Deutsches Herzzentrum der Charité, Campus Virchow Klinikum, Berlin, Germany (A.S.).; 36 Division of Pediatric Cardiology, Duke University Medical Center, Durham, NC (G.F.).; 37 Department of Paediatric Cardiology and Paediatric Intensive Care, University Hospital, LMU Munich, Germany (A.J.).; 38 Department of Pediatric Cardiology, Faculty of Medicine, Hacettepe University, Ankara, Turkey (T.K.).; 39 Pediatric Cardiology Department, Hadassah Medical Center, Jerusalem, Israel (G.M.).; 40 The Heart Institute, Children’s Hospital of Colorado, University of Colorado Hospital, Denver, (G.J.M.).; 41 Department of Pediatric Cardiology, Faculty of Medicine, Izmir Katip Çelebi University, Turkey (N.N.).; 42 Department of Pediatrics Heart Institute, Cincinnati Children’s Hospital, University of Cincinnati College of Medicine, OH (S.S.).; 43 Division of Cardiology, Department of Medicine, University of Washington Medical Center, Seattle (Z.L.S.).; 44 Division of Pediatric Cardiology, Stead Family Children’s Hospital, University of Iowa, Iowa City (O. Aldoss).; 45 Kosuyolu Heart, Research and Education Hospital, Department of Cardiology, Istanbul, Turkey (E.A.).; 46 Congenital Cardiology, Congenital Heart Center, Spectrum Health Helen DeVos Children’s Hospital, Grand Rapids, MI (O. Aregullin).; 47 Service de Cardiologie–CHU Grenoble Alpes, France (H.B.).; 48 Department of Congenital Heart Disease and Pediatric Cardiology, University Heart Center Freiburg–Bad Krozingen, Medical Center, University of Freiburg, Germany (T.F.).; 49 CHRU de Lille, University Lille Nord-de-France, Faculté de Médecine, Institut Cœur Poumon, Service des Maladies Cardiovasculaires Infantiles et Congénitales, Lille, France (F.G.).; 50 M3C-Necker, Hôpital Universitaire Necker-Enfants malades, Assistance Publique- Hôpitaux de Paris (AP-HP), Paris, France (S.M.-M.).; 51 Interventional Cardiology, Home Hospital, Brasilia, Brazil (P.M.).; 52 University Hospital Ramon y Cajal, Madrid, Spain (A.S.-R.).; 53 Imaging and Intervention in Congenital and Structural Heart Disease, Ignacio Chavez National Institute of Cardiology, Mexico City, Mexico (J.P.S.).; 54 Adult Congenital Heart Disease, UT Southwestern, Dallas, TX (W.T.).; 55 Blalock-Taussig-Thomas Pediatric and Congenital Heart Center, Johns Hopkins Children’s Center, Baltimore, MD (J.T.).; 56 Hospital Fornecedores de Cana de Piracicaba, São Paulo, Brazil (P. Tome).; 57 Department of Pediatrics, The Smidt Heart Institute, Cedars Sinai Medical Center, Los Angeles, CA (E.M.Z.).

**Keywords:** atrial septal defect sinus venosus, stents

## Abstract

**BACKGROUND:**

Covered stent correction for a sinus venosus atrial septal defect (SVASD) was first performed in 2009. This innovative approach was initially viewed as experimental and was reserved for highly selected patients with unusual anatomic variants. In 2016, increasing numbers of procedures began to be performed, and in several centers, it is now offered as a standard of care option alongside surgical repair. However, covered stent correction for SVASD is not recognized by regulatory authorities, and in the minds of many pediatric and adult congenital cardiologists and surgeons, the condition is viewed as treatable only by cardiac surgery with cardiopulmonary bypass.

**METHODS:**

In April 2023, all centers identified from international conferences, publications, and colleague networks to be undertaking covered stent correction for SVASD were invited to participate in a retrospective audit of their procedures.

**RESULTS:**

Data were received on 381 patients from 54 units over a 12-year period with 90% of procedures being performed over the past 5 years. Balloon-expandable stents (8 types) were used in the majority; self-expanding stents (4 types) were used in 4.5%. The commonest stent was the 10-zig covered Cheatham Platinum stent in 62% of cases. In 10 procedures, the stent embolized requiring surgical retrieval and repair of the defect, resulting in technically successful implantation in 371 of 381 (97.4%). Major complications (surgical drainage of tamponade, pacemaker implantation, surgery for pulmonary vein occlusion, and late stent removal) occurred in 5 patients (1.3%). Repeat catheterization to correct residual leaks was required in 7 patients (1.8%). Thus, 359 of 381 patients (94.2%) had successful correction without major complications or additional catheter interventions.

**CONCLUSIONS:**

This article details the exponential uptake of covered stent correction for SVASD during the past 5 years. Cardiopulmonary bypass was avoided in the majority of patients, and major complications were infrequent. Prospective registries with standardized definitions, inclusion criteria, and follow-up and comparative studies with surgery are now required to help support the extension of covered stent correction as an alternative standard-of-care option for patients with an SVASD.

**Clinical Perspective**

**What Is New?**

- There has been an exponential increase in the number of covered stent corrections in patients with a sinus venosus atrial septal defect over the past 5 years.
- In this international registry, the success rate of stent implantation in 381 patients was 97.4%, with major complications in 1.3% and repeat catheter interventions for residual leaks in 1.8%.
- In high-frequency units, covered stent correction is currently offered routinely as an alternative to surgery in patients with a sinus venosus atrial septal defect.

**What Are the Clinical Implications?**

- The high success rate and low complication rate of covered stent correction in patients with a sinus venosus atrial septal defect suggest that prospective comparative studies with surgery are now indicated.
- Covered stent correction in patients with a sinus venosus atrial septal defect may come to rival surgical repair as the standard of care.

**Editorial, see p 757**

Covered stent correction (CSC) for a sinus venosus atrial septal defect (SVASD) was first performed in 2009 and briefly alluded to in an article describing anatomic variations of the defect in 2011.^1^ The first detailed case report was published in 2014, and an earlier oral presentation from 2013 was formally published in 2020.^2,3^ Subsequently, several further case reports were published that used different stents.^4–6^ In 2016, significant numbers of procedures began to be performed in 2 centers and were published in 2020; in 2021, data on 75 procedures using covered Cheatham Platinum (CCP) stents were published.^7–9^ In several centers, CSC is currently regarded as a standard-of-care option routinely offered to anatomically suitable patients as an alternative to surgical repair. CSC for SVASD is, however, not recognized by any regulatory authorities, and no stents repurposed or newly developed for this procedure have received approval. In the absence of randomized comparisons,^10^ in the minds of many pediatric and adult congenital cardiologists and surgeons, the condition is viewed as treatable only by cardiac surgery with cardiopulmonary bypass. This article informs on the uptake of CSC from contributors to the SVASD CSC Registry to provide real-world data on the success rates and complications from multiple institutions using a variety of stents.

## Methods

All centers known to be undertaking CSC with any type of stent were invited to join the registry. Centers were identified from participants in the original 10-zig CCP Registry,^9^ conference proceedings, publications,^1–24^ and stent manufacturer databases and through colleague networks. Invitations were issued in April 2023; preliminary results were presented at the Congenital Structural Valvar Heart Disease Interventions conference in June 2023; and data were accepted until August 31, 2023. Data were anonymized; each center identified their patients by a center code and number and the age in years and months. Demographics included age, weight, height, symptoms, and magnetic resonance imaging (MRI) findings. Procedural details included the procedure date, type and size of stent(s), implantation technique, and procedure duration.

### Inclusion

Patients with an SVASD who had no additional reasons for cardiac surgery (valve repair, coronary revascularization, etc) were assessed for anatomic suitability with cross-sectional imaging (computed tomography or MRI) supplemented by 3-dimensional reconstruction.^11^ When the drainage of the anomalous right pulmonary veins to the left atrium was predicted to be adequate after covered stent placement in the superior vena cava (SVC) and there were no high nondivertible pulmonary veins amenable to surgical redirection, the patient proceeded to cardiac catheterization. Final assessment for suitability was made during balloon interrogation of the defect. A balloon was inflated in the SVC to occlude the defect so that the pulmonary venous pathway to the left atrium could be assessed by transesophageal echocardiography (TEE), angiography, and pressure measurements. When the pulmonary venous pathway was confirmed to be unobstructed, CSC proceeded.^7–9^ Patients who were unsuitable on cross-sectional imaging or at cardiac catheterization (pulmonary venous pathway obstruction or inability to eliminate the shunt during balloon interrogation) or who were not considered for CSC because of patient or physician preference were excluded from this retrospective audit.

### Definitions

CSC was considered to be technically successful after stent implantation without embolization requiring cardiopulmonary bypass to remove the stent and correct the defect. Major complications were those requiring additional cardiothoracic surgery. Moderate complications were those requiring intraprocedural interventions, vascular access site interventions, or cardioversion. Minor complications were those that required medication alone or resolved without treatment. Additional catheter interventions were catheterization procedures performed after the initial procedure. Early complications were those occurring intraprocedurally or during the initial hospital stay up to 30 days. Late complications occurred after the hospital stay and beyond 30 days to 1 year. Complications included procedure-specific and general catheterization complications occurring immediately and during follow-up to the census date of August 31, 2023. Residual leaks on angiography or echocardiography were qualitatively described as trivial, small, or moderate, with moderate leaks possibly needing a further procedure.

### Statistics

The Fisher exact test was used to test differences between groups.

The audit was approved by the audit committee of Guy’s & St Thomas’ NHS Hospital Trust. Individual institutional approval was not mandated but was obtained at the discretion of the submitting centers. Results are presented in accordance with the Strengthening the Reporting of Observational Studies in Epidemiology cohort reporting guidelines. The checklist is provided in the Supplemental Material.^25^ Data that support the findings of this study are available from the corresponding author on reasonable request.

## Results

### Data Capture

**Figure 1.**
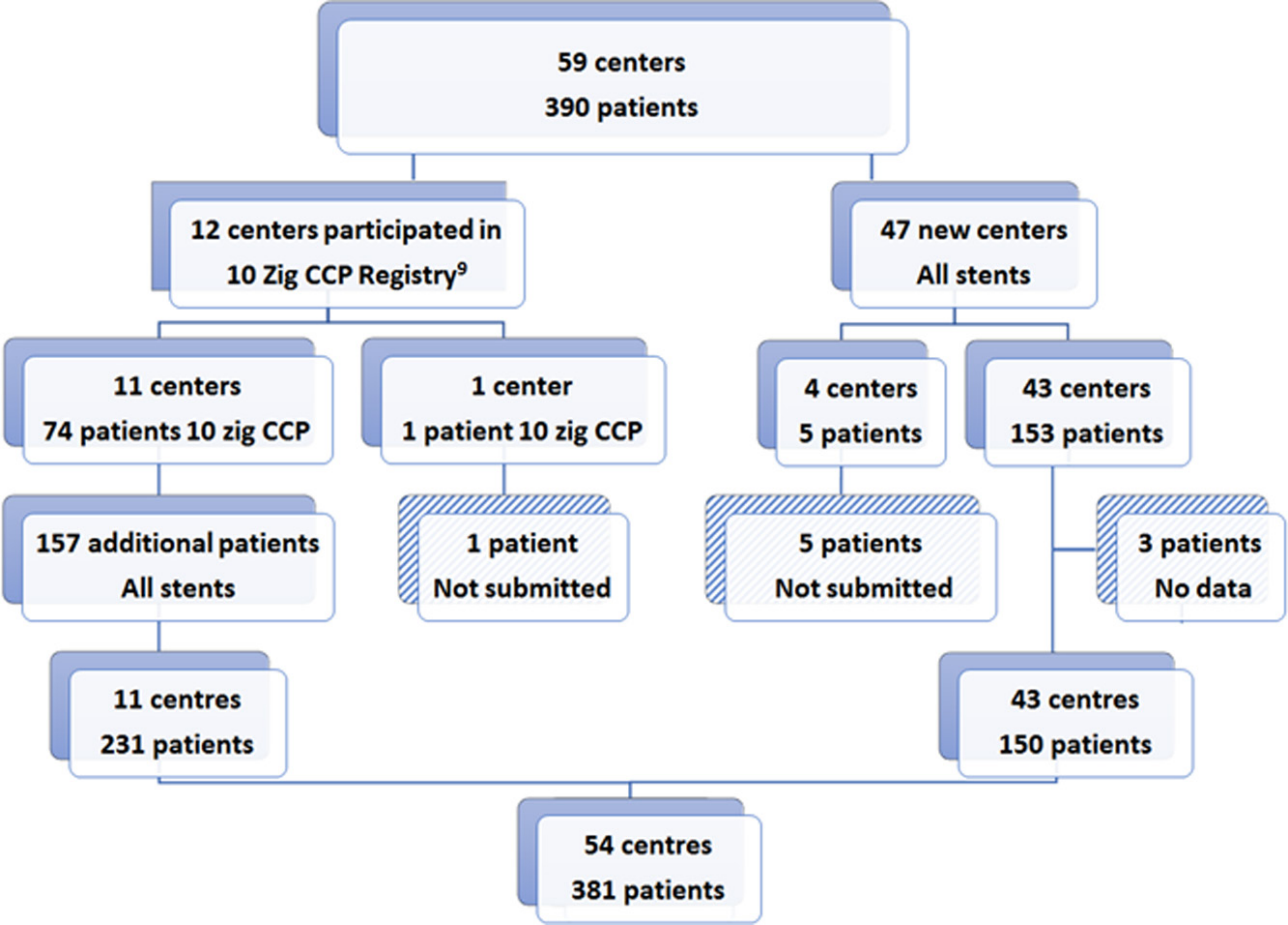
**Study enrollment.** Study enrollment flowchart showing the majority of procedures were undertaken by 11 of 12 of the centers from the original 10-zig Covered Cheatham Platinum (CCP) Registry.^9^ The 43 new centers contributed 39% of the procedures. Of 390 known patients, data were submitted on 381.

Of the 12 centers in the 10-zig CCP registry,^9^ all but 1 participated, extending follow-up and adding new patients. The 12th center could not obtain institutional review board permission and withdrew their single patient. Of the additional 47 centers invited to participate, data were submitted by 43 centers; institutional approval was not obtained in time at 3 centers (4 patients), and one institution (1 patient) failed to respond. One center could obtain data only from its primary hospital but not its secondary hospital (2 patients) because of institutional review board constraints, and one center could not track data on one previous patient after a personnel change. Thus, of a potential 59 centers, 54 submitted data on 381 patients with 3 missing patients, and 5 centers with 6 patients did not participate. Data were analyzed on 381 of 390 known procedures (97.8%; Figure 1).

### Patient Details

The age at the time of the procedure ranged from 4.75 to 87 years (median, 44 years) with a weight of 17 to 145 kg (median, 70 kg). Thirty-seven patients were ≤18 years of age, of whom 14 were 4.75 to 10 years of age (Table 1). Symptoms were present in 307, absent in 65, and not stated in 9 patients. Having >1 symptom was common. In 2 patients, previous surgical repair had left a residual shunt; one was identified when a postoperative pacemaker was needed.^11^ A nondivertible accessory right upper pulmonary vein was present in 72 patients (18.9%), and bilateral SVCs were present in 58 patients (15.2%). Shunt calculations (cardiac catheterization or MRI) confirmed a left-to-right shunt in 198 patients. In only 1 patient was there a right-to-left shunt.

**Table 1.**
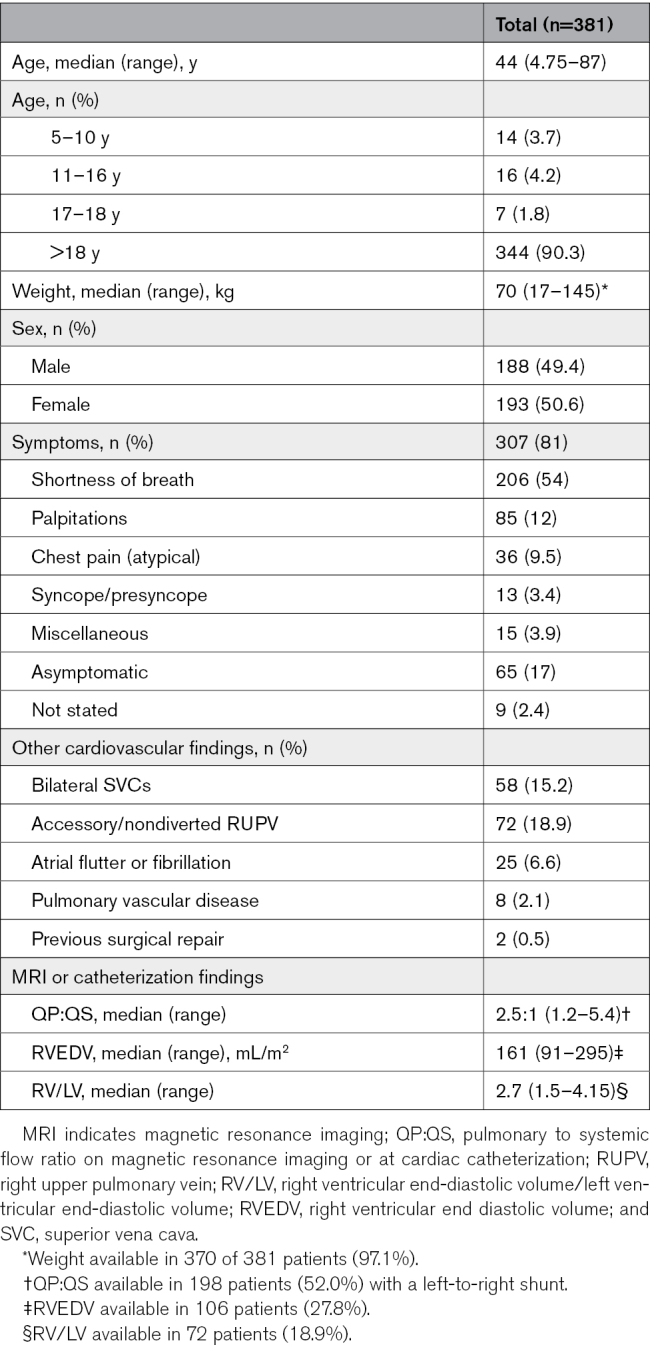
Patient Details

### Procedural Uptake

**Figure 2.**
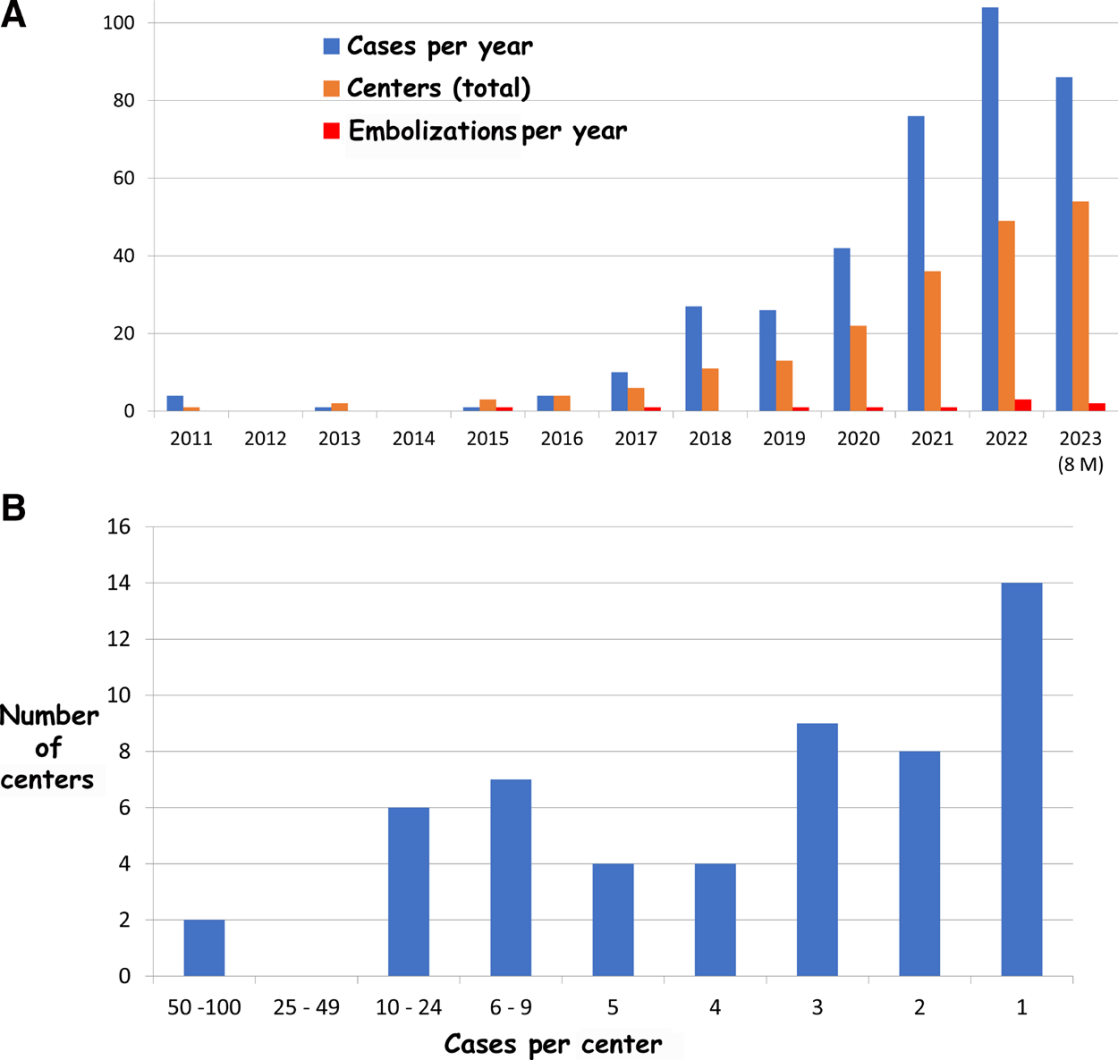
**Number of procedures by year and by center.** The yearly number of procedures is shown by the blue bars; total number of centers undertaking procedures is shown by the orange bars in **A**. Only 8 months of data are available in 2023. The number of cases and centers is shown in **B**.

**Figure 3.**
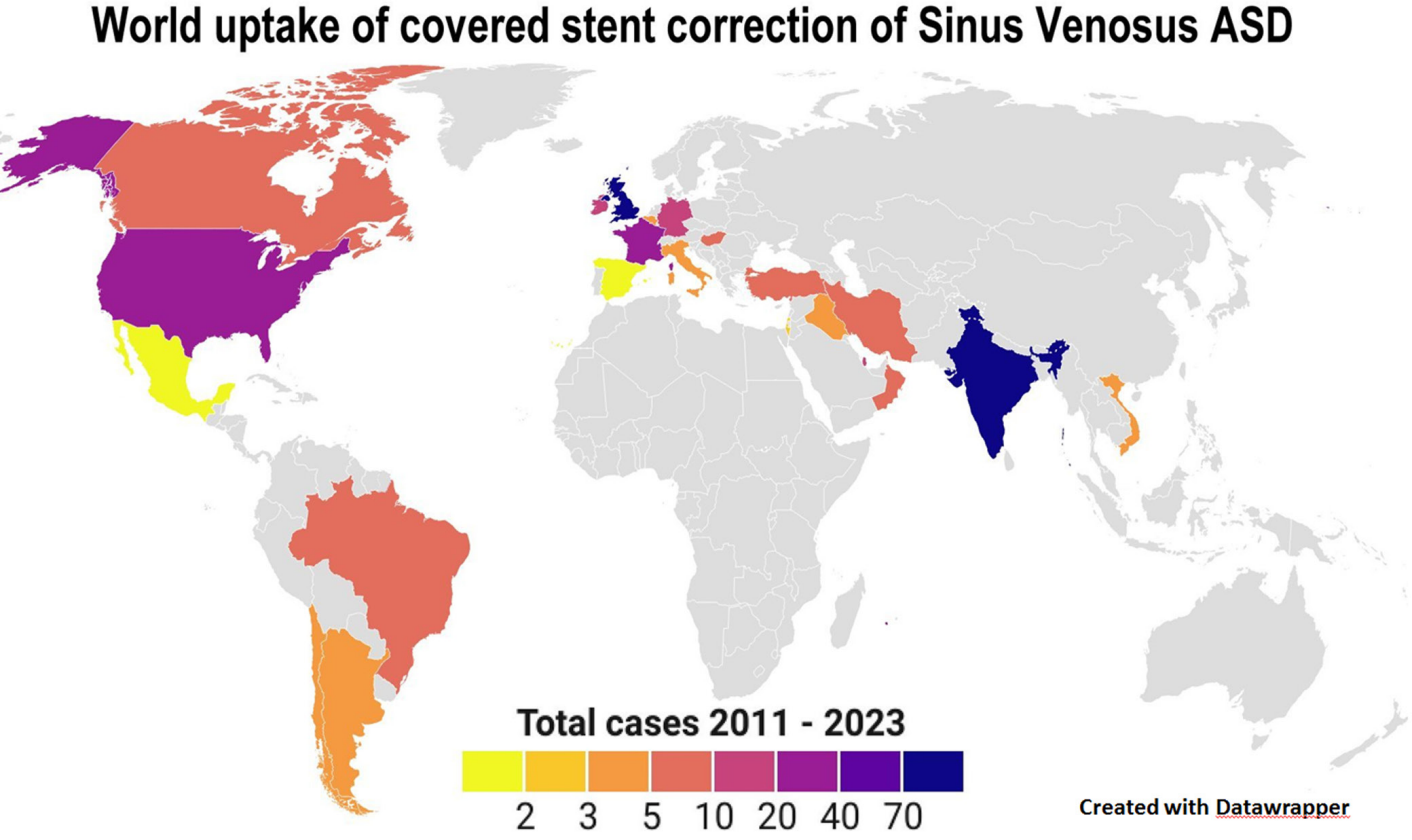
**World uptake of covered stent correction for sinus venosus ASDs.** The color-coded graphic shows the world uptake of the procedure and the large parts of the world that have not yet undertaken procedures (https://datawrapper.dwcdn.net/0BIJU/1/). Actual case numbers are given in Table S1. ASD indicates atrial septal defect.

The first procedure was in 2011, and the last was on August 31, 2023, with <5 procedures annually until 2016 followed by a year-on-year increase such that since 2021 >70 procedures have been performed annually (Figure 2A). During the first 8 months of 2023, >80 procedures were reported, suggesting that the final tally will significantly surpass the preceding years. Mirroring the increased procedure frequency was an increase in centers undertaking CSC. In 2 centers, >50 procedures were performed, whereas 14 centers had undertaken only one procedure (Figure 2B). The largest number of procedures was in India (Figure 3), where balloon-expandable stents were used in 103 of 117 patients, and the United Kingdom, where balloon-expandable stents were used in all 77 patients. The highest crude rate per million of the population was in Qatar at 6.7; only Oman, Ireland, and the United Kingdom had >1 procedure per million of the population (Table S1).

### Stents

**Figure 4.**
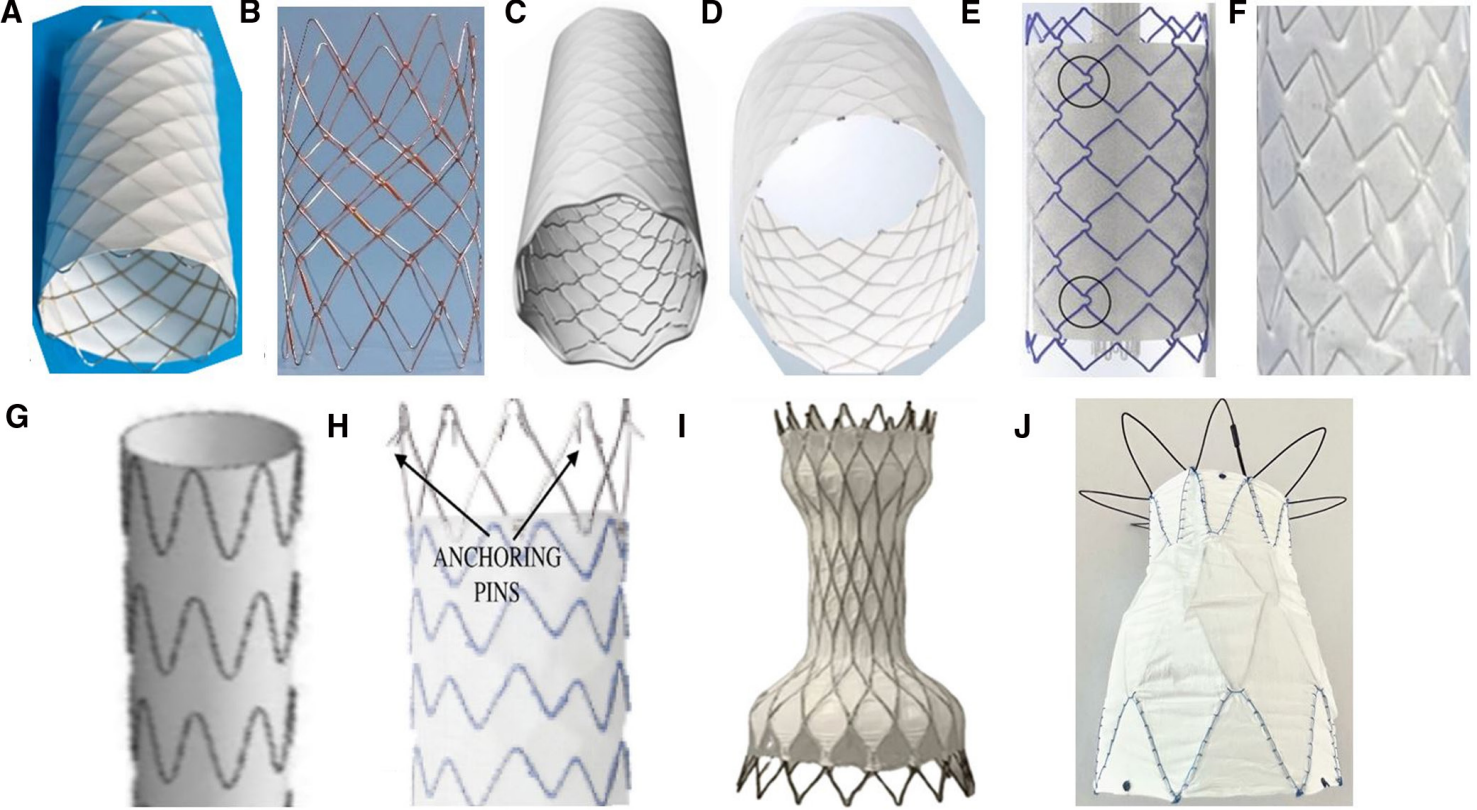
**Stents.** Stents used included 10-zig covered CP (Numed Inc, Hopkinton, NY; **A**),^9^ *10-zig G-Armor (uncovered in illustration) stent (Numed; **B**),^16^† B-graft (Bentley InnoMed GmbH, Germany; **C**),^26^‡ covered Andrastent (Andramed GmbH, Reutlingen, Germany; **D**),^27^§ Optimus Covered Stent (AndraTec GmbH, Koblenz, Germany; **E**),^28^‖ Zephyr covered stent (Sahajan and Laser Technology Ltd, India; **F**),^29^¶ Zenith Flex (Cook Medical, Bloomington, IN; **G**),^30^# Endurant Stentgraft (Medtronic, Minneapolis, MN; **H**),^22^** VB Stent (HeartX, Galway, Ireland; **I**),^18^†† and Nano Self-Expanding Stent (Nano, Florianopolis, Brazil; **J**). The 8-zig versions of **A** and **B** are not shown. *Modified from Rosenthal E, Qureshi SA, Jones M, Butera G, Sivakumar K, Boudjemline Y, Hijazi ZM, Almaskary S, Ponder RD, Salem MM, et al. Correction of sinus venosus atrial septal defects with the 10 zig covered Cheatham-platinum stent: an international registry. *Catheter Cardiovasc Interv*. 2021;98:128–136.^9^ †Modified from Morgan GJ, Zablah J. A new FDA-approved stent for congenital heart disease: first-in-man experiences with G-ARMORTM. *Catheter Cardiovasc Interv*. 2022;100:1261–1266.^16^ ‡Modified from Bentley receives CE mark for new BeGraft aortic stent graft. Vascular News. https://vascularnews.com/bentley-receives-ce-mark-for-new-begraft-aortic/.^26^ Modified from Andramed GmbH. https://andramed.com.^27^ ‖Modified from Haddad RN, Hascoet S, Karsenty C, Houeijeh A, Baruteau AE, Ovaert C, Valdeolmillos E, Jalal Z, Bonnet D, Malekzadeh-Milani S. Multicentre experience with Optimus balloon-expandable cobalt-chromium stents in congenital heart disease interventions. *Open Heart*. 2023;10:e002157.^28^ ¶Modified from Sagar P, Puthiyedath T, Sivakumar K. First-in-man use of an Indian-made balloon-expandable covered Zephyr stent and intermediate-term follow-up. *Ann Pediatr Card*. 2023;16:48–51.^29^ #Modified from Illig KA, Ohki T, Hughes GC, Kato M, Shimizu H, Patel HJ, Shahriari A, Mehta S; Zenith TX2 Low Profile Study Investigators. One-year outcomes from the international multicenter study of the Zenith Alpha Thoracic Endovascular Graft for thoracic endovascular repair. *J Vasc Surg*. 2015;62:1485–1494.^30^ **Modified from Yalamanchi R, Sivaprakasam MC, Janke RVR, Chandrasekharan K, Sadhasivam VS, Showkathali R. Unanticipated complication of transcatheter correction of superior sinus venosus atrial septal defect. *J Cardiol Cases*. 2021;25 99–102.^22^ ††Modified from Vettukattil J, Subramanian A, Barthur A, Mahimarangaiah J. Transcatheter closure of sinus venosus defect: first-in-human implant of a dedicated self-expanding VB stent system. *Catheter Cardiovasc Interv*. 2023;102:1088–1094.^18^

The different stents are illustrated in Figure 4. Balloon-expandable stents (8 types) were used in 364 of 381 cases (95.5%).^3,7–9,12–16^ The majority of centers (33) used only balloon-expandable stents of one type, but 15 used >1 type (Table 2). A mixture of balloon-expandable and self-expanding stents was used in 4 centers, and only self-expanding stents were used in 2 centers.^4,17,18^ The 10-zig CCP stent (Numed Inc, Hopkinton, NY) was the commonest stent (237/381, 62%); 19 centers used only this stent.

**Table 2.**
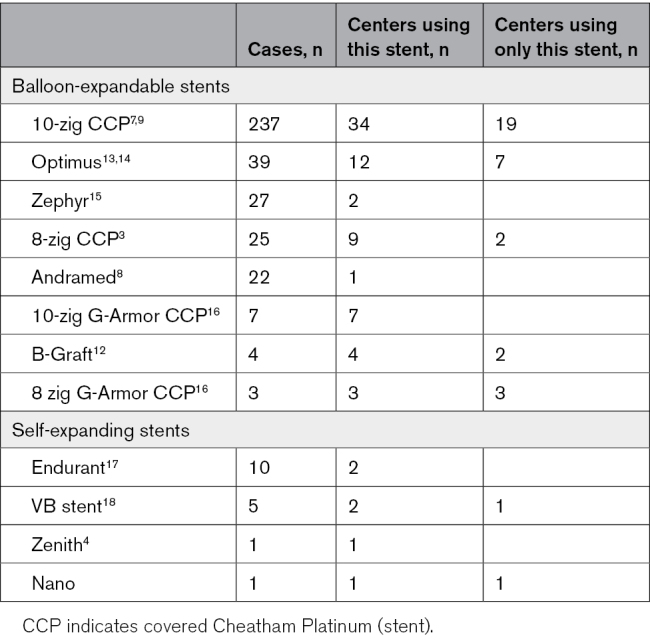
Stent Details

Additional stents (1.8 stents per procedure) were used in 180 procedures (47.2%; Table 3). Anchoring the covered stent in the SVC because of actual or threatened instability was the commonest reason. Additional covered stents sealed residual shunts, usually at the right atrium or SVC end of the stent and less commonly for a midstent endoleak.^19^ Overlapping stents were mounted on one longer balloon in 17 cases because of unavailability of a longer stent or when combined with an uncovered stent to avoid blocking small very high veins above the covered stent^8^ (Figure S1). Two stents were sutured together to form a longer initial stent in 3 cases; the stents separated in 2 cases, requiring an overlap stent in the middle.^20^

### Implantation Techniques

The femoral venous approach for balloon-expandable stents was predominantly over a femoral to internal jugular vein guide wire rail, with occasional placement of the guide-wire tip in a brachial vein. A single stent was used this way in 187 patients (49.1%). Preliminary landing-zone stents were placed in the SVC before siting the covered stent in 74 procedures (19.4%); a third locking stent in the SVC “sandwiched” the covered stent in 33 of these procedures (8.7%; Table 3).^9^ A suture threaded through a zig at the cranial end of the stent and exiting the jugular vein sheath to control stent positioning was used in 48 patients (12.6%).^9,13,21^ Self-expanding stents were used in 17 patients (4.5%): from the femoral vein in 12 and the jugular vein in 5.^4,17,18^ A single stent was used in 14 patients. A landing-zone stent was placed in 3 patients (balloon-expandable stent in 2 and self-expanding stent in 1), and in 1 patient, a third self-expanding stent was needed.^4^

**Table 3.**
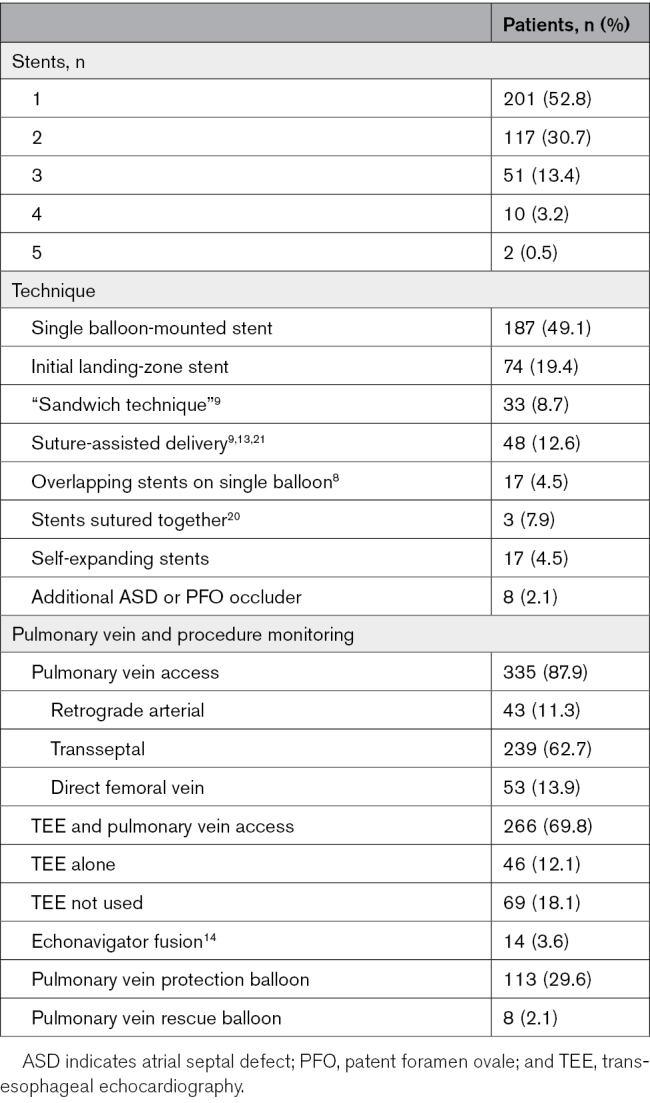
Procedure Details

A concomitant secundum atrial septal defect was closed with an atrial septal defect occluder in 8 patients.^15^ In one patient, transvenous pacing leads were extracted, and new ones were sited immediately after CSC.

### Procedure Monitoring

TEE monitoring was used as an adjunct to pulmonary vein access in 266 (69.8%), used as the sole monitoring technique in 46 (12.1%), and not used in 69 procedures (18.1%; Table 3). Pulmonary vein access to assess suitability and monitor the procedure (pressure and angiography) was obtained in 335 procedures (87.9%). Transseptal access was used in 239 (62.7%) through a transseptal puncture in 166 (43.6%) or a patent foramen ovale or secundum atrial septal defect in 73 (19.2%). A retrograde femoral arterial approach was used in 43 procedures (11.3%), and both antegrade and retrograde access was used in 5 procedures.^7,8^ Direct access from the femoral vein through the SVASD with removal of the pulmonary vein catheter before, during, or after stenting was performed in 53 procedures (13.9%).

### Pulmonary Vein Protection

A balloon was inflated in the pulmonary vein after transseptal access in 113 cases (29.6%) to prevent pulmonary venous pathway compromise during stent implantation^7^ (Figure S2). The pulmonary vein protection balloon deflated inadvertently during stent implantation in 2 cases. In one case, reinflation of the pulmonary vein balloon re-established pulmonary venous flow (Figure S2). In the other, this went unnoticed, and pulmonary venous obstruction caused hemoptysis requiring a thoracoscopic partial upper lobectomy at 3 months.^9^ In 8 cases (2.1%), obstruction of the pulmonary venous pathway occurred without previous pulmonary vein protection, and subsequent balloon inflation in the pulmonary vein molded the covered stent to allow unobstructed pulmonary venous pathway flow.^14^

### Procedural Outcome

Stent implantation was technically successful in 371 of 381 procedures (97.4%). In 10 patients (2.6%) at 8 different centers, the stent embolized to the right atrium or right ventricle. All underwent surgical retrieval and standard surgical repair with uneventful recovery. This was intra-procedural in 6 patients and occurred after leaving the catheter laboratory or by the next day in in 4 patients. Most occurred during the first 6 procedures at 7 different centers and continued to occur throughout the study period as new centers joined (Table 4; Figure 2A). Embolization occurred with balloon-expandable stents in 9 of 364 (2.5%), with self-expanding stents in 1 of 17 (5.9%), after an initial landing-zone stent in 3 of 77 (3.9%), with the suture holding technique in 1 of 48 (2.1%), and in children in 1 of 37 (2.7%). There was no significant difference in the frequency of embolization based on stent length (7/176 with stents <7 cm long and 3/205 with stents ≥7 cm long).

**Table 4.**
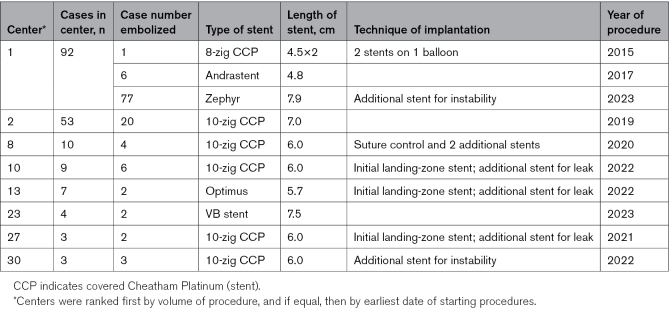
Embolization of Covered Stent/s

Immediately after the procedure, there was no leak in 194 (51%), a trivial leak in 121 (31.8%), a small leak in 36 (9.4%), and a moderate leak in 7 (1.8%) with no comment in 13 (3.5%). High nondiverted veins were left draining above the level of the covered stent in 22 patients, were through a bare metal anchoring stent in 23 patients (Figure S1), were covered over in 12 patients without apparent sequelae, and were not recorded in 15 patients. Procedural times (n=312, 81.9%) ranged from 50 to 450 minutes (median, 143.5 minutes) with fluoroscopy times (n=305, 80.1%) of 9.31 to 123 minutes (median, 33.4 minutes).

### Complications

#### Early Complications

There were 5 major (1.3%) and 39 moderate and minor (10.2%) complications and 7 catheter reinterventions for residual leaks (1.8%). The combined incidence was 4 of 37 (10.8%) in children and 47 of 344 (13.7%) in adults.

Early complications are shown in Table 5.

##### Major Complications

Cardiac tamponade in 2 patients required surgical drainage in the first week. The transseptal puncture was assumed to be the cause in one patient.^7^ The anchoring hooks of a self-expanding stent penetrated the aorta in another and were covered with pledgets.^22^

Sinus node dysfunction developed in one patient hours after the procedure, necessitating a pacemaker 3 days later. At 1 year, there were signs of sinus node recovery.^23^

##### Moderate and Minor Complications

The majority of moderate and minor complications resolved during the procedure or during the hospital stay.

Innominate vein obstruction in 2 patients from stents placed too high in the SVC resolved with balloon dilation in one patient after needle puncture of the covered stent.^8^ Pulmonary venous pathway narrowing was relieved with intraprocedural balloon dilation in 8 patients (2%).

Intraprocedural thrombus in 6 patients (1.57%) resolved with intraprocedural heparin.

In terms of access site, a significant jugular hematoma resolved uneventfully, but a femoral hematoma underwent surgical exploration. A superficial femoral puncture site infection responded to antibiotics. A self-expanding stent delivery mechanism was trapped by a second stent and removed by jugular vein cut down. In 4 patients, a femoral artery pseudoaneurysm responded to compression and thrombin injections.

Atrial fibrillation in 2 patients reverted with cardioversion. One patient developed atrial flutter that was cardioverted 2 days later.

Pulmonary hemorrhage due to a guide-wire injury required intensive care but resolved over 2 weeks in one patient. One patient had a minor pulmonary vein dissection without extravasation, and stent implantation proceeded uneventfully.

Pericardial effusions resolved with conservative management in one patients: in one patient by day 3 and in one patient after a course of colchicine.

Prolonged procedures and poor patient positioning led to a neuropraxia in 4 patients that resolved completely over several weeks.

In-stent thrombus was detected in 1 patient and resolved after the antiplatelet agents were changed to anticoagulation during the in-hospital stay.

#### Late Complications

Late complications are shown in (Table 5).

**Table 5.**
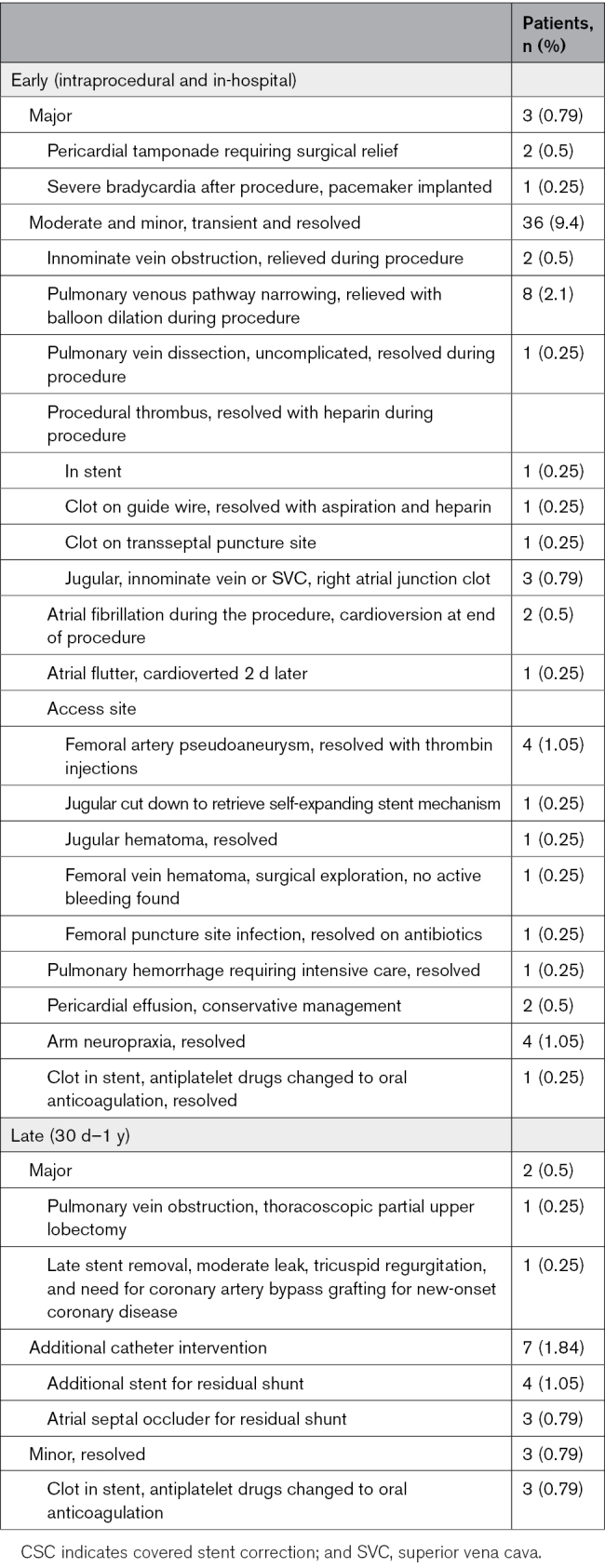
Complications After Successful CSC

##### Major Complications

Pulmonary vein obstruction of an accessory right upper lobe pulmonary vein caused ongoing hemoptysis in one patient, who underwent thoracoscopic partial upper lobectomy at 3 months.^9^

A patient with a moderate residual shunt, tricuspid regurgitation, and newly detected coronary artery disease had surgical correction of all his defects with stent removal at 1 year.

##### Additional Catheter Intervention

Significant residual shunting required an additional stent or atrial septal defect occluder during the first year in 7 patients (1.8%).

##### Minor Complications

In-stent thrombus was detected in 3 patients by routine TEE in the weeks after the procedure. These resolved after changing anti-platelet agents to anticoagulation.^24^

### Deaths

There was no procedural mortality, but 3 patients died after successful procedures. A 60-year-old man in congestive heart failure with pulmonary hypertension had a cerebral bleed (mechanism not ascertained), developed pneumonia, and died 3 weeks after the procedure. A 73-year-old woman died of a brain tumor >12 months after the procedure. A 70-year-old man died 2 years after the procedure of complications of Crohn disease.

### Follow-Up

At many centers, follow-up was rarely available for more than a postoperative visit with subsequent follow-up at the referral centers, especially when the patient had traveled from abroad. Follow-up ranged from 1 day to 12.4 years with a median of 1.7 years. The leak had resolved in an additional 44 of 121 with a trivial leak, 12 of 36 with a small leak, and 3 of 7 with a moderate leak. The 1-year follow-up was still awaited for 118 patients. A formal MRI shunt calculation was available in 51 patients (13.4%), documenting a fall in pulmonary to systemic flow ratio from 2.5±0.68 to 1.2±0.3.

One patient had a pacemaker for asymptomatic pauses on Holter monitoring, but it was unclear whether this predated CSC 14 months previously.

## Discussion

CSC for SVASD was initially viewed as an experimental approach in highly selected patients with unusual anatomic variants but unlikely to be applicable to the majority of patients.^1–6^ Over the past few years, there has been an exponential rise in the volume of procedures and number of units offering CSC.^7–24^ This registry included data from 54 centers with their respective learning curves over 12 years. CSC was successful in 371 of 374 patients (97.4%) and free of major complications or repeat catheter interventions in 359 of 381 patients (94.2%). The majority of moderate and minor complications resolved or were corrected during the procedure or in-hospital stay and were typical of general cardiac catheterization procedures. The relatively long procedural times of an unfamiliar procedure during the learning curves contributed to their occurrence.

### Stenting Technique

A range of stents and implantation techniques were used. One larger center used 5 different balloon-expandable stents; another used only a single type. Newer and longer stents (custom length 10-zig CCP, Optimus, G-Armor) with less shortening (Zephyr) are increasingly used and should reduce the risk of complications and residual leaks. “Innovative” strategies to elongate a stent by suturing 2 stents together are no longer needed. Overlapping covered and uncovered stents to avoid occluding high nondivertible pulmonary veins can be replaced with purpose-designed stents that are partially covered. Although attractive conceptually and frequently discussed in interventional meetings, self-expanding stents were used far less frequently than anticipated. Nevertheless, newer self-expanding stents continue to emerge and may yet have a role in specific anatomic subsets (VB, Nano).

Monitoring during the procedure also varied considerably. Transesophageal monitoring was used most frequently, commonly accompanied by pulmonary vein monitoring. The majority of operators used transseptal access for pulmonary vein monitoring to facilitate pulmonary venous pathway protection when needed or as a rescue route for pathway obstruction after stent implantation. Some centers, in carefully selected patients, avoided this, thereby simplifying the procedure considerably. That balloon dilation of the pulmonary venous pathway after stenting was still occasionally required suggests that pulmonary venous access needs to be considered carefully when the pulmonary venous pathway appears borderline and when new operators start CSC. It is important to emphasize that although ostensibly a straightforward procedure once the anatomy is understood and the concept is accepted, it remains technically challenging. Most embolization occurred during the first 6 procedures undertaken in a unit, but there was also late embolization in the 2 most experienced centers. New operators and centers may benefit from observing procedures and guidance from proctors.

### Comparison With Surgery

As with many new procedures that develop in the face of an established technique, CSC for SVASD has not been formally compared with surgical repair. Avoidance of sternotomy and cardiopulmonary bypass with a short hospital stay (the majority of patients go home the day after the procedure and return to normal activities and work within a few days) is a major advantage of CSC. Particularly in the developing world, lower costs and length of hospital stay would benefit many.

Complications reported after surgical repair (residual defects, SVC and pulmonary vein stenosis, and sinus node dysfunction) vary between centers and with surgical techniques. One-patch, 2-patch, and the Warden repairs have their proponents and different complication profiles and have modifications that differ across centers. The Warden procedure can cause SVC obstruction, requiring reoperation or stent implantation in as many as 22%.^31^ Sinus node dysfunction ranges from 14% to 55% after the 2-patch repair, depending on the era and center.^32–34^

The impetus to perform comparative studies depends on evidence of noninferiority and potential superiority that is slow to accrue with small case reports and limited series. Modifications during the learning curve and experience with different stents in different centers also impede this by introducing variations. This registry highlights that CSC using a range of stents and techniques successfully avoided the morbidity of open cardiac surgery in the vast majority. Minor and resolvable catheter complications were not infrequent during the learning curves of multiple institutions but are anticipated to decrease as the technique becomes established; for example, femoral pseudoaneurysms (1%) are avoidable if retrograde catheterization of the pulmonary vein is replaced by direct or transseptal access, and arm neuropraxia (1%) should be reduced with shorter procedures as the technique becomes familiar. Stent embolization in 2.6% required cardiac surgery with cardiopulmonary bypass, but this is anticipated to decrease with greater experience with the procedure and the availability of longer and newer stents. The incidence of stent embolization was too low to confirm that longer stents embolized less frequently, and data on the length of stent apposition to the SVC as a possible cause of embolization were not available.

Two patients in this registry had residual shunts after surgical repair and underwent successful CSC. Crossover of patients between the modalities highlights the interdependence of congenital interventional cardiology and cardiac surgery. A small, nonrandomized, era-based comparison showed similar rates (and different types) of complications, but the output of this registry indicates that larger, formal, prospective studies should now be considered.^10^ Such studies in patients with secundum ASDs have shown shorter hospital stay, lower costs, and fewer complications in the catheter closure group compared with the surgical group.^35–37^

### Uptake of the Procedure

This registry highlights the uneven uptake of CSC. Although the crude rates per million of population do not take into account equity of health opportunities, socioeconomic factors, health tourism, and population movements, it is apparent that large parts of the world have not engaged with this procedure and that many patients are not considered for it. Currently, many pediatric and adult congenital cardiologists and surgeons still view the SVASD as a wholly surgical condition. The aim of the registry was to inform practitioners of our specialty on the current state of CSC, and the registry has now collected data showing upward of 100 procedures annually in the past 2 years and upward of 54 centers that now offer the procedure. The number of centers and procedures is likely to continue to rise despite the absence of formal regulatory approval, which is surely needed now. The absence of either US Food and Drug Administration approval for the procedure itself or CE marking for any custom made stents currently in use is an important obstacle to wider uptake.

### Limitations

Although identification of centers, as described, may not have included every center undertaking CSC, it is likely that the majority were invited. Only a few centers with small numbers of patients did not submit data, thus 98% of known procedures were included. Data collection accuracy depended on the goodwill of the participants, as did the reporting of complications. The indications for SVASD closure, procedural approach, stent implantation techniques, anticoagulation regimen, and follow-up were not standardized in this retrospective study. Neither was the definition of residual shunting by either TEE or angiography at implantation or by TEE, transthoracic echocardiography, computed tomography, or MRI scanning during follow-up. Routine TEE was obtained at one large and several smaller centers in the first 1 to 6 months, whereas another large center performed a computed tomography scan at 2 months and an MRI scan at 1 year. Many centers discharged patients to local care after the first follow-up. The absence of rigorous quantification of residual shunts and documentation of the timing of complete closure is the most limiting aspect of this study, although typically this is not performed after surgical repair currently. Unlike surgery, during which high draining veins can be diverted with patches or the Warden procedure (although in some cases, they are left behind), these veins are typically left draining to the SVC during CSC. Quantification of the amount of residual shunting through these smaller veins was not undertaken, although the overall shunt in those with follow-up MRIs was negligible. Sinus node function was not systematically evaluated before or after the procedure. The single acute instance of sinus node dysfunction requiring a pacemaker after CSC was considerably lower than that seen after surgery,^26–28^ and the sinus node dysfunction appeared to recover during further follow-up.^23^ Although 9.7% of the patients were children, further data and longer follow-up are required to assess fully the effects of growth. Last, the proportion of patients who were considered unsuitable for CSC is unknown and should be addressed in future studies.

### Conclusions

CSC for SVASD was performed in 381 patients at 54 units over a 12-year period with >90% in the past 5 years. Cardiopulmonary bypass was avoided in the majority of patients, and complications were infrequent. Longer, newer stents and evolving techniques are likely to reduce the incidence of complications and residual shunts further and streamline the procedure. Therefore, CSC has moved from being a restricted investigational option in rare, highly selected patients to an increasingly commonly performed procedure. Indeed, in several centers, when the anatomy appears favorable, it is now offered as a standard-of-care option alongside surgical repair. Prospective registries with standardized definitions, inclusion criteria, and follow-up and comparative studies with surgery are required before CSC can be recommended more widely as an alternative option to surgical repair.

## Article Information

### Acknowledgments

The authors are grateful to the following for support of this project with data collection and procedural assistance: Neha Ahluwalia, MD, Children’s Hospital of Michigan, Detroit Medical Center, Detroit, MI; Felix Berger, MD, Department of Congenital Heart Disease–Pediatric Cardiology, Deutsches Herzzentrum der Charité, Berlin, Germany; Mario Carminati, MD, IRCCS Policlinico San Donato, Milano, Italy; Ivan Casserly, MD, Mater Misericordiae University Hospital, Dublin; Peter Ewert, MD, Department of Congenital Heart Diseases and Pediatric Cardiology, German Heart Center, Munich, Germany; Marc Gewillig, MD, Division of Congenital and Structural Cardiology, UZ Leuven, Belgium; Albenque Grégoire, MD, Department of Congenital Heart Diseases, Hospital Marie Lannelongue, Paris, France; Nikolaus Haas, MD, Kinderkardiologie und Pädiatrische Intensivmedizin, Klinikum der Universität München, München, Germany; Dr Raymond N. Haddad, M3C-Necker Enfants Malades, AP-HP, Paris, France; Berhan Keskin, MD, Kosuyolu Heart, Research and Education Hospital, Department of Cardiology, Istanbul, Turkey; Brian Morray, MD, Cardiac Catheterization Laboratories, Seattle Children’s Hospital, Seattle, WA; Dao Anh Quoc, MD, University Medical Center, Ho Chi Minh City, Vietnam; Kolja Sievert, MD, CardioVascular Center Frankfurt, Frankfurt, Germany; Murat Sürücü, PhD, Department of Pediatric Cardiology, Dr. Siyami Ersek Hospital for Cardiology and Cardiovascular Surgery, Istanbul, Turkey; and Mark Turner, MD, Adult Congenital Cardiology, Bristol Heart Institute, Bristol, UK.

### Sources of Funding

None.

### Disclosures

Drs Morgan, Qureshi, and Hijazi are consultants for Numed. Drs Levi and Steinberg are consultants for B. Braun. Drs Fraisse and Kempny are consultants for AndraTec. Dr Rosenthal is a proctor for BVMedical. The other authors report no conflicts.

### Supplemental Material

Supplemental Methods

Table S1

Figures S1 and S2
